# Wing morphology predicts individual niche specialization in *Pteronotus mesoamericanus* (Mammalia: Chiroptera)

**DOI:** 10.1371/journal.pone.0232601

**Published:** 2020-05-11

**Authors:** Hernani Fernandes Magalhães de Oliveira, Nícholas Ferreira Camargo, David R. Hemprich-Bennett, Bernal Rodríguez-Herrera, Stephen J. Rossiter, Elizabeth L. Clare

**Affiliations:** 1 Department of Ecology, Evolution, and Organismal Biology, Iowa State University, Ames, Iowa, United States of America; 2 School of Biological and Chemical Sciences, Queen Mary University of London, London, England, United Kingdom; 3 Laboratório de Ecologia de Vertebrados, Universidade de Brasília, Brasília, Brazil; 4 Department of Zoology, University of Oxford, Oxford, England, United Kingdom; 5 Escuela de Biología, Universidad de Costa Rica, San José, Costa Rica; Uppsala Universitet, SWEDEN

## Abstract

Morphological variation between individuals can increase niche segregation and decrease intraspecific competition when heterogeneous individuals explore their environment in different ways. Among bat species, wing shape correlates with flight maneuverability and habitat use, with species that possess broader wings typically foraging in more cluttered habitats. However, few studies have investigated the role of morphological variation in bats for niche partitioning at the individual level. To determine the relationship between wing shape and diet, we studied a population of the insectivorous bat species *Pteronotus mesoamericanus* in the dry forest of Costa Rica. Individual diet was resolved using DNA metabarcoding, and bat wing shape was assessed using geometric morphometric analysis. Inter-individual variation in wing shape showed a significant relationship with both dietary dissimilarity based on Bray-Curtis estimates, and nestedness derived from an ecological network. Individual bats with broader and more rounded wings were found to feed on a greater diversity of arthropods (less nested) in comparison to individuals with triangular and pointed wings (more nested). We conclude that individual variation in bat wing morphology can impact foraging efficiency leading to the observed overall patterns of diet specialization and differentiation within the population.

## Introduction

Individuals within a population may show differences in behaviors and/or morphologies that can influence the ways in which they explore, compete, and utilize food resources [1–6]. Such variation may in turn, therefore, lead to niche differentiation and specialization due to individuals having different food preferences, with a consequent expansion of the overall dietary niche [6]. It follows that species that are considered to be generalist may in fact include individuals that are relatively specialized in their resource use [6–7]. More work is needed to examine the potential ecological and evolutionary implications of such variation, including consequences for trophic niche dynamics in natural populations [6, 8].

Inter-individual variation in a morphological trait may allow the exploration of different micro-niches, so potentially reducing intraspecific competition [2, 4]. As a result, these individuals might be expected to show dissimilarity in diet due to individual feeding specializations [9–10], a phenomenon observed across a wide range of invertebrate and vertebrate taxa and different ecosystem [5, 11–12].

Wing shape in bats varies considerably among species, and correlates with differences in flight mode and speed [13–14]. Variation in wing shape is associated with differences in habitat use [14–15], with broad wings and rounded wing tips considered more adapted for slower maneuverable flight, and thus a tendency to forage in cluttered environments such as dense vegetation. Conversely, species characterized by narrower pointed wings are associated with faster flight in open areas, and are less maneuverable [14]. These differences in habitat use could have potential consequences for bat-arthropod interactions, as vegetation density is a good estimator of prey availability for bats, with more cluttered habitats showing higher prey abundance [16–17]. In addition to influencing habitat use, flight maneuverability might also confer advantages in hunting, increasing the probability of successful prey capture [18–21]. To date, most investigations of the variation in bat flight ability, diet and morphology have consisted of comparisons between bat species [14–15] with little consideration given to the impact of variation within populations. Nevertheless, intraspecific variation in wing morphology has been detected in bats, such as the little brown bat (*Myotis lucifugus*; [16]) and the little yellow-shouldered bat (*Sturnira lilium*; [22]). Similarly, *P*. *parnellii* [11] exhibits individual variation in habitat use and diet [12].

Network approaches can provide useful insights into inter-individual variation in resource use [23–24]. The existing studies that have examined networks composed of individuals within a population have reported different levels of specialization [25–26], in which the diets of specialist individuals are nested within the diets of the generalists [7, 27]. Although it has been shown for many species that individual variation in diet is linked with differences in morphological traits [9–10], to understand how trait variation between individuals may explain their position in ecological networks can be relevant to shed light on variation in network structure and individual specialization in natural populations. Such interaction networks perform best when the nodes (taxa) are assigned at the lowest taxonomic level possible, and, as a result, DNA barcoding and metabarcoding have been increasingly advocated as important methods for building ecological networks [28–29]. DNA-based approaches are typically based on molecular operational taxonomic units (MOTUs) [30], which represent clusters of sequences with a minimum similarity threshold. This method does not assign nodes in the network to the species level of resolution [31] but applies a uniform threshold to all nodes in a network making comparisons more standardized [32].

Here we apply network approaches to examine the relationship between wing morphology and diet in the insectivorous bat *Pteronotus mesoamericanus* in the dry forest of Costa Rica. This species was previously classified as a subspecies of *P*. *parnellii*, but has since been elevated to species-level based on genetic and morphological evidence [11]. As with *P*. *parnellii*, it appears to forage over cluttered and non-cluttered habitats, and has potential for individual variation. Our objective was to explore resource use patterns at the individual-level, as determined by the DNA metabarcoding of prey remains within guano pellets of known bats, and relating these dietary data to wing morphological parameters. By characterizing the diets of bats within a network framework, we tested the hypothesis that variation in network position of individual predators (diet specialization and differentiation) is related to differences in individual wing shape. More specifically, we made the following predictions: 1) individuals with rounded wings and thus more maneuverable flight will be able to exploit a wider range of prey items. Therefore, they would have a more generalist diet with a higher number of food items (prey MOTUs) and be associated with less nested positions in the network. On the other hand, individuals with more pointed wings and less maneuverable flight will explore a narrower range of MOTUs (i.e, narrow niche widths), and thus, would have a more specialist diet and higher nested positions in the network; 2) wing shape is a good predictor of individual differentiation in niche use. Variation in wing shape among individuals will be associated with increasing diet dissimilarity, due to the exploration of different resources that reflect differences in hunting efficiencies and the possible exploration of different habitats.

## Materials and methods

### Study area

The study was conducted in dry forests of Costa Rica, located at Sector Santa Rosa (10°46.7´N, 85°39.8´W) in the Área de Conservación Guanacaste (ACG), which is composed of primary and secondary forests in different stages of regeneration [33]. It experiences large variations in precipitation due to its extreme seasonal climate [34–35], with a dry season (December to May) when there is virtually no rain, and a wet season, with an annual precipitation of 900 to 2,400 mm [33, 36]. During our study there was a strong El Niño (2015) when the park experienced an unusually low annual rainfall of only ~600 mm.

### Bat sampling, diet and network analysis

We sampled bats during the wet season (Jul—Aug) of 2015. We captured bats using four to six mist nets (6 m– 12 m x 2 m) opened from 18h–22h along trails and near watercourses in three locations of similar habitats within a 2 km range of distance from the main camp site. Bat tissue was sampled by wing membrane biopsies as part of a different simultaneous study and thus we were able to assess whether individuals were recaptured by the presence of a hole or scar. Each bat was held in a cloth bag for a maximum of two hours to collect faecal samples and each sample was stored in 70% ethanol and frozen (-20°C). Faecal samples only reveal the composition of diet over a short period of time before they were collected since gut retention times in insectivorous bats are generally less than 90 minutes [37]. Each individual was identified to species following Reid [38]. All bats were captured and handled according to the recommendations of the American Society of Mammalogists [39]. Research was performed under permit R-07-2015-OT-CONAGEBIO and R-08-2015-OT-CONAGEBIO, from the Ministry of Environment and Telecommunications (MINAET) and Comisión Nacional para la Gestión de la Biodiversidad (CONAGEBIO).

### DNA extraction, PCR amplification and sequencing

We extracted DNA from faecal samples using the QIAamp Stool Mini Kit (Qiagen, UK) following manufacturer’s instructions with the modifications suggested by [40] and [41]. Amplification, gel electrophoresis and amplicon size selection, clean up and sequencing were all performed at the Biodiversity Institute of Ontario, University of Guelph (Canada). PCR Primers based on the COI primers ZBJ-ArtF1c and ZBJ-ArtR2c were used to amplify prey DNA [40]; these primers were modified using the dual adaptor system for the Ion Torrent [41]. Each 20μL PCR reaction contained 10μL of Qiagen multiplex PCR (Qiagen, CA) master mix, 6μL of water, 1μL of each 10μM primer and 2μL of DNA. PCR amplification was as follows: 95°C, 15 min; 50 cycles of 95°C, 30 s; 52°C, 30 s; 72°C, 30 s and 72°C, 10 min. Amplicons were visualized on a 2% agarose 96-well precast E-gel (Invitrogen, Life Technologies). Size selection was performed using a PCRClean DX kit (Aline Biosciences). The product was eluted in water, and the concentration measured using a Qubit 2.0 spectrophotometer and the Qubit dsDNA HS Assay Kit (Invitrogen, Life Technologies). The products were normalized to 1ng /μL prior to final library dilution. Sequencing was performed on the Ion Torrent (Life Technologies) sequencing platform as per [41] using a 316 chip and following the manufacturers’ guidelines but with a 2x dilution.

### Data analysis

We processed sequences using the Galaxy platform [42–44]. We de-multiplexed the samples by forward and reverse MIDs (a maximum of two mismatches and two indels were allowed) and removed primer, MID and adapter sequences (http://hannonlab.cshl.edu/fastx_toolkit). We filtered out all sequences shorter than 147 bp or longer than 167 bp (target amplicon length was 157bp) and collapsed them into unique haplotypes and then excluded singleton sequences from further analyses (http://hannonlab.cshl.edu/fastx_toolkit). We clustered sequences into molecular operational taxonomic units (MOTUs) and picked representative sequence of each MOTU for analysis with the QIIME pick_otu and uclust methods (http://qiime.sourceforge.net/, [45]). For the present study MOTUs were clustered using a similarity threshold of 92%. This is a more conservative approach for the generation of the numbers of MOTUs [32]. We repeated the analyses at 94 and 96% MOTU thresholds to confirm that the obtained results were not dependent on bioinformatics choices. These additional analyses can be seen in S1 File.

We used a single representative sequence per MOTU for order-level identification using BLAST analyses and a database of >600,000 reference DNA barcodes extracted from GenBank. We used MEGAN version 5.6.3. [46] to screen out unidentified sequences and those not resolved to level of taxonomic order with the LCA parameters: Min score = 150.1, Max expected = 0.01, Top percent = 10.0, Min support = 1, LCA percent = 10.0, Min complexity = 0.2. We looked for chimeric sequences from each reference sequence using UCHIME as implemented in MOTHUR [47], and for contaminants by looking for similar BLAST matches to nontarget taxa (e.g. bacteria) in MEGAN (with the same parameters as above). The identified MOTUs were used for network analysis. We then generated datasets at 94% and 96% MOTU thresholds to repeat analyses and confirm that outcomes were not dependent on bioinformatics choices (see below and S1 File).

### Geometric morphometric analysis

**Fig 1:**
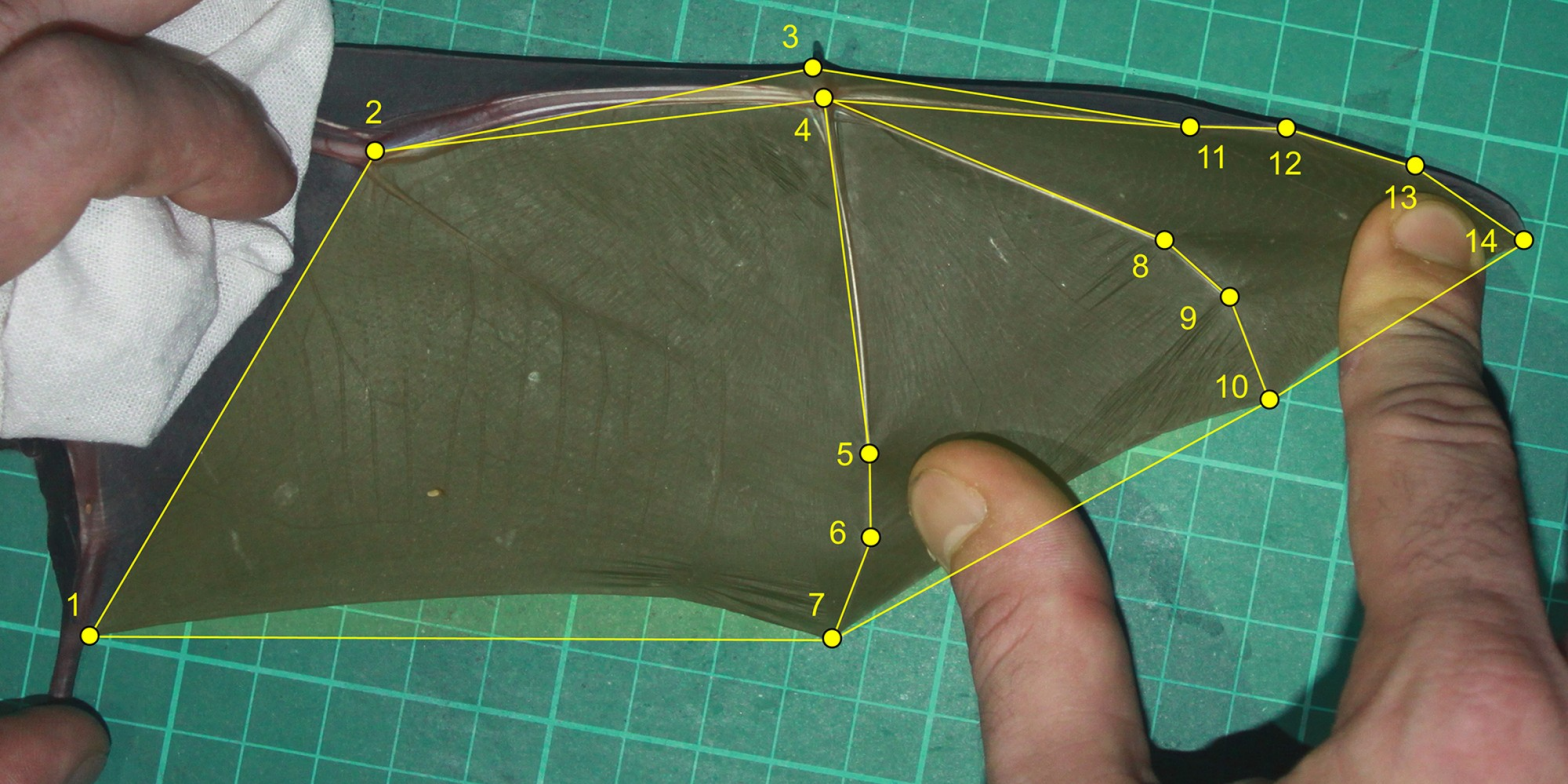
Photograph showing the method by which the bat wing was stretched in order to record the following anatomical landmarks. 1) Tissue junction between the wing and the hind foot; 2) Articulation between the humerus and radius/ulna; 3) Tissue junction between the propatagium membrane and digit I; 4) Center of the carpus; 5) Articulation between metacarpus and proximal phalange of digit V; 6) Articulation between proximal and distal phalanges of digit V; 7) Tissue junction between distal phalange of digit V and propatagium membrane; 8) Articulation between metacarpus and proximal phalange of digit IV; 9) Articulation junction between proximal and distal phalanges IV; 10) Tissue junction between distal phalange of digit IV and dactylopatagium major membrane; 11) Articulation between metacarpus and proximal phalange of digit III; 12) Articulation between proximal and intermediate phalanges of digit III; 13) Articulation between intermediate and distal phalanges of digit III; 14) Tissue junction between distal phalange of digit III and dactylopatagium medius membrane.

We performed geometric morphometric analyses from information collected from bat wing photos of *P*. *mesoamericanus* adults. To photograph specimens, the left wing of each individual was extended against a grid with 1 cm marking guides and photographed with a digital camera (Canon EOS DIGITAL REBEL T1i—Canon EF-S 18–55 lens), which was mounted on a tripod at a fixed height (80 cm). For the standardization of wing position, we have considered the fifth finger parallel to the body of the animal and the largest possible stretching of major (digits IV and V) and medius (digits III and IV) dactylopatagium membranes (Fig 1). Additionally, we considered the maximum angulation between the humerus and the radius/ulna (Fig 1). The arm extension of bats is related to the stretch capacity of the propatagium membrane. Therefore, if there was any resistance in the extension of the bat’s arm, to avoid injury, we have considered this as the maximum angulation between humerus and radius/ulna. For each individual, we extended and photographed its wings three different times for further evaluation of the standardization of the method. Wing images were only taken from adult males and non-pregnant females in order to avoid biases in the wing shape of juveniles and sub-adults due to the incomplete development of their wings, and to avoid stress to pregnant females since their diet may vary due to their physical condition. For the evaluation of the wing shape of *P*. *mesoamericanus*, we selected 14 anatomical landmarks with the support of the software TpsDig v.1.4 [48]. Anatomical landmarks were used as a way to sample homologous portions of the wing, and were represented only by tissues joints (phalanges, cartilage and wing membrane; Fig 1).

To quantify potential error related to the standardization method used to extend the bat wings before obtaining the images, we checked the repeatability of anatomical landmarks measurements in 20 individuals (eleven males and nine females) using the three photos taken from each of them. For this, we used the intraclass correlation coefficient from an analysis of variance on the x and y coordinates of each anatomical landmark. From this analysis we were able to verify the error in locating the anatomical landmark position and the differences between individuals. We confirmed that all landmark locations were highly repeatable [49] across samples, with intraclass correlation coefficients ranging from 0.95 to 0.99 (experimental error between 0.02 and 0.020 pixels; variance between 0.12 and 0.83 pixels). Thus, we assumed that the method adopted to extend the wings was standardized throughout the study. Mean variation in measurements for any one individual was 81.2 pixels while mean variation between individuals was 791.0 pixels.

After obtaining the 14 landmarks for each analyzed individual, we obtained the wing shape variables (partial warps and uniform components) from the superimposition of anatomical landmarks (Procrustes algorithm) using the software TpsRelW v.1.62 [50]. This method involves the centralization and minimization of distances between anatomical landmarks and the standardization of anatomical landmarks configuration from the Centroid Size (CS) [51–52]. The CS is a multivariate measurement of size of the structure analyzed, which is is obtained by the square root of the sum of the square distance of each anatomical landmark to the mass center of each configuration (centroid) [51].

### Network analysis

In order to assess how specialized each individual bat was with respect to diet, we compiled the observed interactions into a presence (assigned as 1) and absence (assigned as 0) matrix with each cell value representing the interactions between each individual pair (individual bat and insect MOTU). Differences in individual niche use (interaction specialization) were assessed in the network using values of nestedness. In this case, nestedness is a measure of the level to which the interactions are specialized or generalized [53]. To quantify nestedness values for each individual, we used the function nestedrank with the binmatnest algorithm from the Bipartite R package [54] which can specifically accommodate unweighted data matrices. Nestedrank rearranges the network of interactions according to its maximal possible nestedness and then quantifies the level of specialization of a given node (individual bat) through its rank in the matrix [55]. Nestedness values range from 0 to 1, and individuals with high values indicate that they are more specialized.

### Statistical analysis

#### Wing morphology

To identify significant changes in wing morphology between individuals, we performed a Principal Component Analysis (PCA) using the wing shape variables (i.e., partial warps and uniform components). For further analyses, we obtained two new variables (PC1 and PC2) that summarized most of the variation (>60%, see Results for more details) of the wing shape. To visually evaluate the variation in wing shape across individuals within these two axes, we used the software TpsRelW v.1.62. For evaluating any sexual dimorphism in the wing shape, we performed a Hotelling T^2^ test using the PC1 and PC2. For this analysis, we considered each sex as an independent variable and the scores of each individual obtained in the PCs as dependent variables. Additionally, to investigate any size-related bias in the wing shape of *P*. *mesoamericanus* (i.e., allometry), we performed simple regressions using individual scores of each PC as dependent variables and the centroid size of each individual as an independent variable.

#### Relationship between wing morphology and diet

To quantify the similarity of the diet among individuals, we generated a new matrix containing the different food items pooled by arthropod order, based on the presence absence matrix described above (see network analysis section). For example, if individual bat 1 had consumed 8 MOTU assigned to Coleoptera, this individual was assigned a Coleoptera frequency of 8. Then we performed a Principal Coordinate Analyses (PCoA) using the Bray-Curtis dissimilarity index in the vegan package [56] in R [57]. To test for the relationship between variation in wing shape and individual specialization in diet, we performed a multiple regression between the individual scores obtained in the first two principal components (summarized wing shape as independent variables) and the values of individual nestedness (dependent variable). Similarly, to assess how much of the difference between individual diet (diet dissimilarity) could be related to differences in wing shape, we performed a multiple regression between the first two axis of the PCA (PC1 and PC2) (independent variables) and the first axis of the PCoA (dependent variable). For this analysis, we selected only the first axis of the PCoA because it represented a relatively high percentage (about 61%) of the total variance of the individuals’ diet (see S1 File for more details).

We ran all statistical analysis using the R statistical language and environment [57].

## Results

### Diet description and network analysis

**Fig 2:**
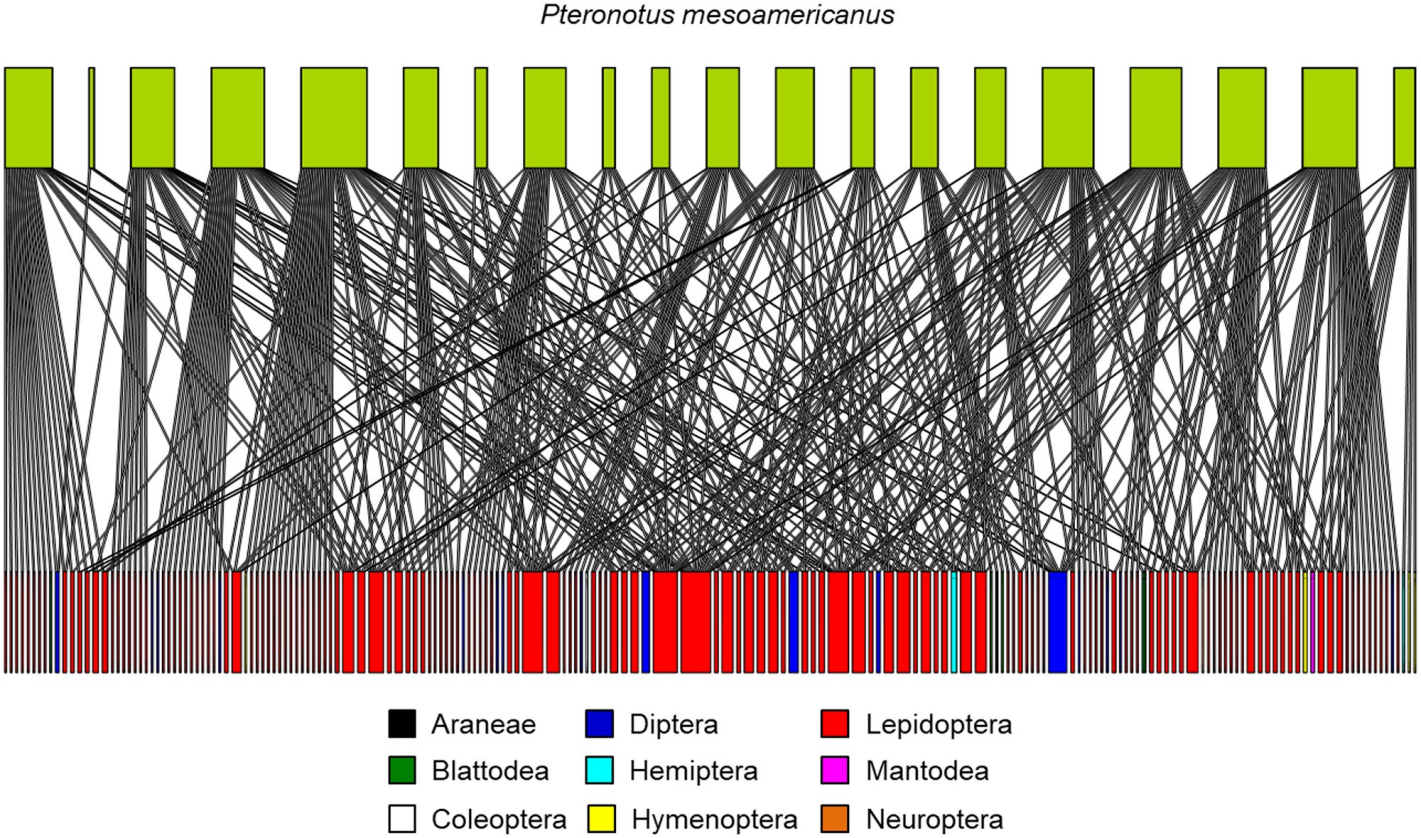
Antagonistic network of individuals of the bat species *Pteronotus mesoamericanus* and the prey items present on their diets. Links in the network representing species diets were revealed using DNA metabarcoding (gene COI). The width of the top bars represent the number of feeding items present in the diet of an individual while the width of the bottom bars represents the number of individuals that consumed that prey item.

We analysed the diet of 20 individuals of *P*. *mesoamericanus* for which we were able to obtain data on wing morphology, spanning a time range of 20 days to limit the effect of temporal variation in prey. Their diet was composed of nine arthropod orders, with Lepidoptera recorded as the most diverse order with 152 MOTUs, followed by Diptera with 16 MOTUs, and Hymenoptera and Blattodea with four MOTUs each (Fig 2). Individuals consumed from three to 36 MOTUs (mean±standard deviation; 19.6±9.0) of one to five orders (2.5±1.2), with Lepidoptera recorded as the most prevalent order present in the diet of all individuals (See S1 File for a description of the diet). Values of nestedness from the ecological network showed extreme variation ranging from 0.00 (indicating an extreme generalist diet composed of a large number of arthropod MOTUs) to 0.95 (indicating an extreme specialist diet composed only of few arthropod MOTUs). However, on average individuals had an intermediate level of diet specialization (nestedness, 0.47±0.30) (See S1 File for additional information).

### Wing morphology

The analysis of the landmarks using the program TpsRelw generated 24 shape variables. The first axis of the PCA (PC1) using these variables explained 34.2% of the total wing shape variance. The main modifications on the wing shape included the displacement of the landmarks 2–13 towards the upper left side of the wing when considering positive score values of the PCA. This component can be interpreted as morphological changes in shape related with wing bulging, especially considering the landmarks 3 and 4 (first digit and the carpus) and 11–14 (third digit). Additionally, this component was also related to changes on plagiopatagium width (wing space between the body and fifth finger) due to the displacement of the landmarks 5–7. The second axis (PC2) explained 26.7% of the total wing shape. This component is related to the displacement of the landmarks 2–14 towards the bottom right side of the wing when considering positive score values of the PCA. The PC2 axis be interpreted as an overall elongation of the wing. Both components together represented more than 60% of the total wing shape variance. Even though each component represented a different variation on wing shape, low PC scores from both components generally suggested more triangular and pointed wings while higher scores were associated with more rounded wings. We found no differences in the wing shape between males and females (Hotelling T^2^_2,17_ = 0.62; P = 0.25). Moreover, we found no association between the wing shape and the centroid size indicating no allometric effects considering both PC1 (r^2^ = 0.18; F_1,18_ = 4.02; P = 0.06) and PC2 (r^2^ < 0.01; F_1,18_ = 0.01; P = 0.76).

### Relationship between wing morphology and diet

**Fig 3:**
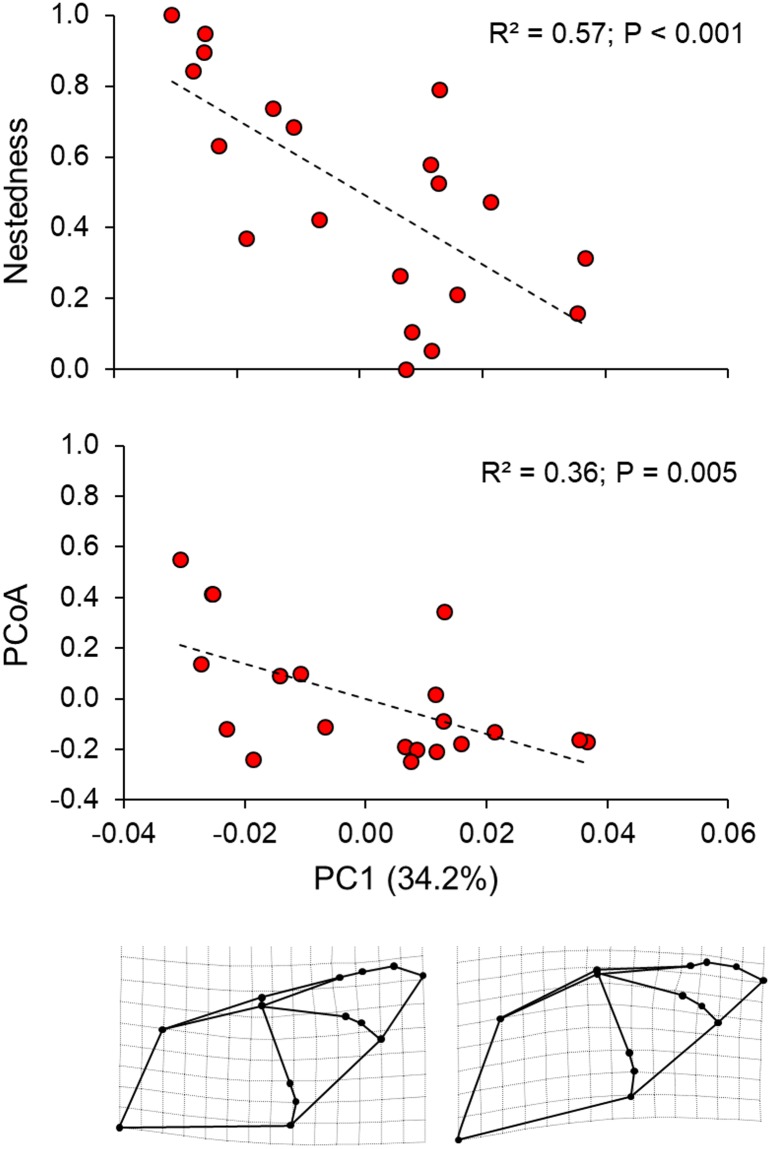
Similarity (PCoA) between wing shape and individual diet of *Pteronotus mesoamericanus*, considering nestedness and similarity (PCoA). The wing shape representations below the x-axis indicated the extrapolated twofold values of the lowest (left inset) and highest (right inset) PC1 scores. Low scores represent wings with a more triangular shape while high scores represent a more rounded wing. The value in parenthesis indicates the proportion of the total wing shape variance; r^2^ and P-values are indicated according to the partial correlation obtained in the multiple regression analysis (global adjusted r^2^ = 0.60 for nestedness and global adjusted r^2^ = 0.40 for PCoA; see results for more details).

**Fig 4:**
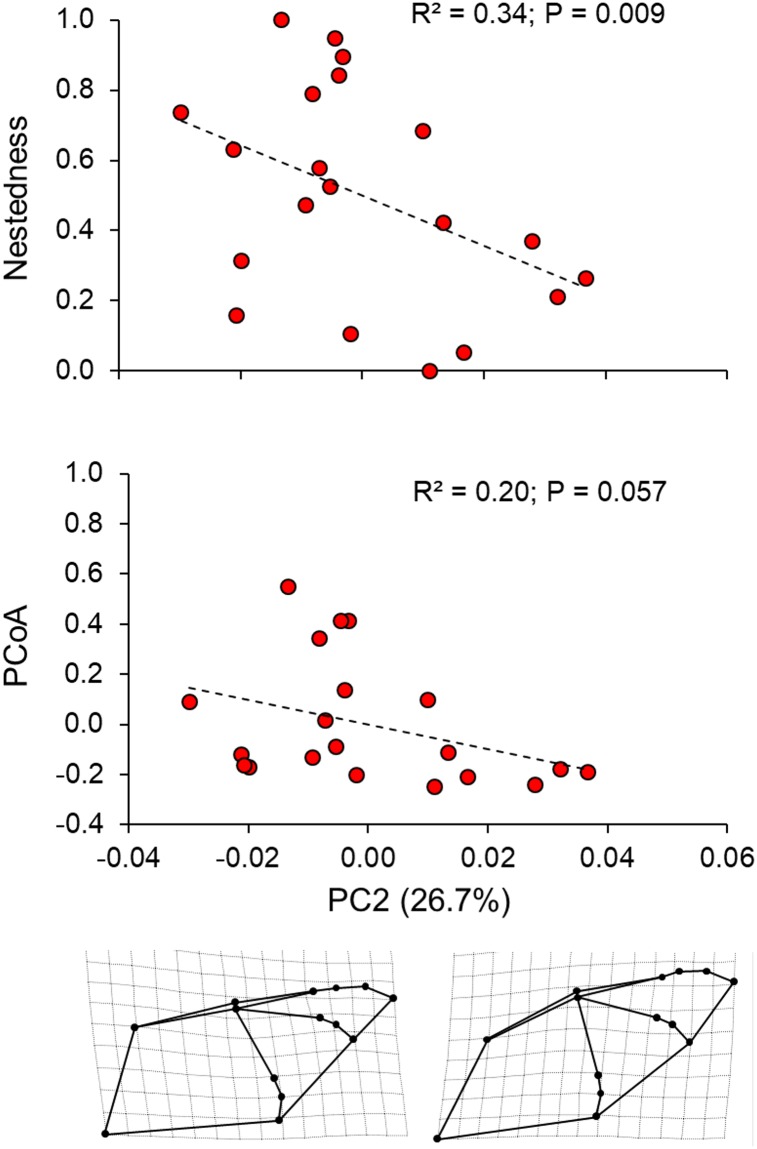
Similarity (PCoA) between wing shape and individual diet of *Pteronotus mesoamericanus*, considering nestedness and similarity (PCoA). The wing shape representations below the x-axis indicate the extrapolated values twofold of the lowest (left inset) and highest (right inset) PC2 scores. Low scores represent wings with a more triangular shape while high scores represent a more rounded wing. The value in parenthesis indicates the proportion of the total wing shape variance; r^2^ and P-values are indicated according to the partial correlation obtained in the multiple regression analysis (global adjusted r^2^ = 0.60 for nestedness and global adjusted r^2^ = 0.40 for PCoA; see results for more details).

The multiple regression analysis demonstrated a significant relationship between wing morphology (PC1 and PC2) and individual values of nestedness from the ecological network (global adjusted r^2^ = 0.60; F_2,17_ = 15.51; P < 0.01) (Figs 3 and 4; Table 1) showing that individuals with pointed wings had a more specialized diet. The analysis between the PCs and diet similarity (first axis of PCoA) also showed a significant association (global adjusted r^2^ = 0.40; F_1,18_ = 7.39; P = 0.005) (Figs 3 and 4) (see S1 File for details), revealing that individuals with similar wing shape also possess similar diets. However, these associations were significant only for PC1 (Table 1). All observations were consistent across MOTU thresholds (see S1 File).

**Table 1:** Partial results for each dependent variable from the two multiple regressions investigating the relationship between wing morphology (PC1 and PC2) and individual specialization (nestedness), and wing morphology and diet similarity (PCoA).

Analysis	Axe	Beta	Partial correlation	T_17_	P-value
Nestedness	PC1	-0.683	-0.754	-4.730	<0.001
Nestedness	PC2	-0.424	-0.581	-2.941	0.009
PCoA	PC1	-0.578	-0.620	-3.259	0.005
PCoA	PC2	-0.362	-0.443	-2.038	0.057

## Discussion

Different individuals within a population can differ in the way that they use resources [58–59], leading to diversification in niche use and a reduction in intraspecific competition through the exploitation of different parts of the environment by individuals [3]. Niche diversification is more likely to happen when the population is formed by individuals with different ecological requirements, where each individual uses a subset of the niche of the whole population [58]. One of the main factors that account for differences in individual niche use is phenotypic variation, which can influence foraging behavior, resource preferences, physiological requirements, and even social status and dominance [60].

In our study, we combined metabarcoding, network analyses and measurements of wing shape to assess the relationship between individual morphological variation and resource use in a population of *Pteronotus mesoamericanus*. In line with our hypothesis, we found that individuals with more rounded wings were associated with greater numbers of MOTUs in their diets, consistent with more generalist diets, than were individuals with more pointed wings. Consistent with this observation, we also found that individuals with more similar wing shapes also shared more similar diets. We observed no differences in the wing shape between males and females, indicating that our findings could not be explained by any sexual dimorphism in wing morphology.

Many previous studies of dietary niche have estimated the degree of specialization as the proportion of food items consumed by an individual in relation to total available in the population [61–63]. However, in most DNA-based metabarcoding studies of environmental, samples, including ones based on guano pellets, prey species (or MOTUs) can only be reliably classified as either present or absent. Moreover, in our study, the difficulty of recapturing wild bats for obtaining repeated measures also precluded the possibility of creating weighted matrices to represent their diets. As a consequence, individual diets in our study are based on presence and absence of prey species recorded in the population as a whole, rather than on the frequency of prey item abundances, and thus we were also unable to derive a number of network metrics (e.g. H’). We therefore measure ‘specialization’ by assessing dietary niche breadth using the relative number of links each individual makes with their prey after reorganizing the matrix of interactions on a nested manner, such that an individual with fewer prey links is ranked by the analysis as more specialized.

Variation in dietary niche breadth might relate not only to different flight capabilities, but also to differences in habitat use that are unrelated to flight, and it is difficult to separate these two possibilities. Bat species with pointed wings are better able to hunt high-flying insects that are more common in open spaces, while bats with rounded wings are better able to hunt insects in the understory [64]. In open spaces, densities of insects, and thus prey availability, tend to be lower, leading to a narrower niche and more specialized diet [17–18]. Considering within-population differences, habitat use related to wing morphology, or wing loading capacity (defined as the total bat body mass divided by the area of its wing), have been reported for at least two bat species. A study conducted with *Myotis lucifugus*, showed that wing loading explained 20% of the variation in habitat use between individuals [17]. Moreover, for *Miniopterus schreibersii*, individuals captured in cluttered environments had shorter wingspans, and lower aspect ratios (defined as wingspan^2^/wing area), than bats captured in open areas [65].

Even though some network metrics have unpredictable changes when ecological networks are constructed with different MOTU thresholds [66], we have used the metrics of nestedness and niche overlap that appear to be resilient to this effect, with only small changes imposed by clustering parameters [32, 66], and the specific threshold used in our study is within the recommended range values (90%-95%) for MOTU determination using these molecular protocols and primers [67]. Our additional analyses, which were run using the MOTU thresholds 94% and 96%, have also shown consistent significant relationships between bat wing shape with diet dissimilarity and specialization (See S1 File). As such, we do not anticipate a strong effect of clustering level on the analyses presented here.

*P*. *mesoamericanus* was originally included in *P*. *parnellii* and was only recently described as a separate species in Central America, distinct from other *Pteronotus* lineages elsewhere in Mexico, the Antilles and South America [12]. Thus, analyses of ecological variation in this taxon are still lacking. *Pteronotus parnellii* has the wing shape of a generalist species with intermediate values of wing loading and aspect ratio in relation to other Neotropical bat species, which makes it possible to exploit different habitats, such as more open and cluttered spaces [68]. Variation in the use of cluttered environments by *P*. *parnellii* is possibly related with insect availability (mass and composition) [13], which might indicate that individual variation in diet is habitat-linked for other species of the *Pteronotus* complex as well. However, until now no study has linked this flexibility to morphological variation among individuals.

Our results suggest that inter-individual morphological variation may lead to different habitat exploitation and different diets among individuals as a consequence. What is still unknown is whether this habitat and resource use partitioning is fixed for each individual within the population regardless prey availability, or whether individuals can exploit alternative niches when food availability is low (e.g., dry season or during El Niño events). For example, in high population densities and consequently low food resources availability, phenotypically different individuals of the fish *Gasterosteus aculeatus* add different alternative prey, increasing individual variation [3].

While morphological features are fixed within an individual bat, echolocation can be highly plastic and in some bat species individuals alter aspects of their echolocation signals with habitat use [69]. Higher frequencies (short wavelengths) are better able to resolve smaller targets, but are more prone to atmospheric attenuation and are more commonly associated with cluttered environments. In contrast, lower frequencies are associated with foraging in more open areas [70]. However, bat species that possess high duty-cycle echolocation, such as some species of the New World genus *Pteronotus*, are less flexible in their calls, and unable to adjust their frequency [71]. If frequency use is less variable in *Pteronotus mesoamericanus*, as may be the case in high-duty cycle bats, inter-individual morphological variation (as measured here) may be much more important in determining individual variation in diet or habitat use.

Studies evaluating different animal groups [5, 28, 72, 73] in highly seasonal ecosystems in the Neotropics have shown that individual variation and specialization are stronger during the resource-rich season (wet season) during which individuals have the opportunity to specialize on different resources [74], generating an overall expansion of the population trophic niche. Optimal Diet Theory (ODT) proposes that resource specialization is dependent on the forager density, prey availability, and variation on forager phenotypes, which act together to determine how different individuals explore, compete, and specialize on their diets [7, 74]. Our study was conducted during the wet season, when there is potentially also population recruitment by reproduction. Under the ODT framework, our scenario with high resource availability, but increased intraspecific competition, would have led to a moderate decrease in resource specialization that would weaken the signal of phenotypical variation on diet specialization and variation, which do not strongly support our findings [7, 74].

On the other hand, in the present study we evaluated individual variation in the wet season during a strong El Niño, and it is likely that this climatic event promoted low abundances of arthropods (D. Janzen, pers. comm.) due to the extreme low precipitation, which could have led to individual ecological release [75]. 2015 was drier in comparison to the previous 31 years in the park. Even though the dry forests of ACG have been suffering an insect decline across the last decades [76], this extreme drought is likely to have decreased arthropod abundances even more. This extremely low resource abundance could also have been one of the main factors that has driven individual specialization due to higher intraspecific competition and diet differentiation in order to avoid competition and increase their survival. We suggest that longer term studies are needed to investigate individual feeding variation in *P*. *mesoamericanus* in both dry and wet seasons, to understand the roles of food resources availability and diet specialization, as well as the consistency of the patterns found in the present study.

Even in the absence of El Niño, dry forests are highly seasonal and show a pronounced difference in habitat structure and insect abundance across seasons [38, 77–79]. Dry forest trees show a seasonal loss of leaves and tree growth [80], which can dramatically change the landscape and forest structure. Variation in wing morphology within the population might enable different individuals to cope with foraging in different habitats (open versus cluttered) across the whole year, including variation in forest structure and prey availability across seasons. However, it is hard to predict how behaviorally flexible individuals with different wing shapes can be during transitions between seasons. Changes in habitat structure and food availability in a Neotropical seasonal dry forest has been linked with differential spatial and resource use between individuals in a mammal species [81]. Thus, it is possible that both variables play a role on how bats with different wing morphologies explore and specialize in their environment.

## Conclusions

Our results indicate that individual morphological variation in wing shape of the bat *Pteronotus mesoamericanus* explains individual variation in diet. Individuals with more triangular and pointed wings were found to be more specialized, whereas individuals with more rounded wings had a more generalist diet and similarly, measures of dietary dissimilarity were related to dissimilarity in wing shape. The individual variation found in the present study may be due to the capability of individuals to access slightly different habitats (open versus cluttered habitats) with different prey availabilities (richness and abundance) and species composition. The present study indicates that changes in wing morphology can play an important role as a source of individual variation and feeding specialization within natural populations of bats. However, other longer term studies are required to assess the consistency of the patterns that we have observed.

## Supporting information

S1 File (DOCX)
