# Supplementary material for: Wing morphology predicts individual niche specialization in Pteronotus mesoamericanus (Mammalia: Chiroptera)
Source: PLoS One. 2020 May 11;15(5):e0232601. doi: 10.1371/journal.pone.0232601 (PMC7213686; doi:10.1371/journal.pone.0232601)
Supplement: S1 File — (DOCX) [file pone.0232601.s001.docx]

**Wing morphology predicts individual niche specialization in *Pteronotus mesoamericanus* (Mammalia: Chiroptera)**

Oliveira, H. F. M.; Camargo, N. F.; Hemprich-Bennett, D. R.; Rodríguez-Herrera, B.; Rossiter, S. J. & Clare, E. L.

**Running title:** Bat wing shape predicts diet differentiation

**Authors:**

Hernani Fernandes Magalhães de Oliveira. Address: Department of Ecology, Evolution, and Organismal Biology, Iowa State University, United States of America / School of Biological and Chemical Sciences, Queen Mary University of London, United Kingdom.

Nícholas Ferreira Camargo. Address : Laboratório de Ecologia de Vertebrados, Universidade de Brasília, Brazil.

David Richard Hemprich-Bennett. Address : Department of Zoology, University of Oxford / School of Biological and Chemical Sciences, Queen Mary University of London, United Kingdom.

Bernal Rodríguez-Herrera. Address: Escuela de Biología, Universidad de Costa Rica, Costa Rica.

Stephen James Rossiter. Address: School of Biological and Chemical Sciences, Queen Mary University of London, United Kingdom.

Elizabeth Lloyd Clare. Address: School of Biological and Chemical Sciences, Queen Mary University of London, United Kingdom.

**Corresponding author:** Hernani Fernandes Magalhães de Oliveira. Address: School of Biological and Chemical Sciences, Queen Mary University of London, United Kingdom. E-mail address: oliveiradebioh@gmail.c

**Table S1**. Results for the Principal Coordinates Analysis (PCoA) evaluating the diet of 20 individuals of *Pteronotus mesoamericanus.* The analysis was performed with the Bray-Curtis dissimilarity index using a matrix containing the different food items pooled by arthropod order for each individual.

| **Analysis** | **Eigenvalue (92%)** | **Variance explained (%) (92%)** | | **Eigenvalue (94%)** | **Variance explained**  **(%) (94%)** | | **Eigenvalue (96%)** | | **Variance explained (%) (96%)** | |  |
| --- | --- | --- | --- | --- | --- | --- | --- | --- | --- | --- | --- |
| PCoA1 | 1.178 | 61.3 | 1.317 | | | 58.76 | | 1.288 | | 55.96 | |
| PCoA2 | 0.344 | 17.9 | 0.286 | | | 12.75 | | 0.352 | | 15.29 | |
| PCoA3 | 0.147 | 07.6 | 0.185 | | | 8.24 | | 0.173 | | 7.53 | |
| PCoA4 | 0.118 | 06.2 | 0.153 | | | 6.84 | | 0.159 | | 6.90 | |
| PCoA5 | 0.063 | 03.3 | 0.078 | | | 3.45 | | 0.100 | | 4.36 | |
| PCoA6 | 0.044 | 02.3 | 0.045 | | | 2.00 | | 0.052 | | 2.25 | |
| PCoA7 | 0.022 | 01.1 | 0.028 | | | 1.26 | | 0.038 | | 1.65 | |
| PCoA8 | 0.06 | 0.3 | 0.020 | | | 0.89 | | 0.018 | | 0.76 | |

| **Table S2.** Consumption of MOTUs from different arthropod orders at the MOTU threshold 92% by individuals of *Pteronotus mesoamericanus* in the dry forests of Costa Rica. Cell values represent the number of Molecular Operational Taxonomic Units (MOTUs) consumed per individual. Nestedness, number of orders and total number of MOTU consumed are indicated. | | | | | | | | | | | | | | | | | | | | |
| --- | --- | --- | --- | --- | --- | --- | --- | --- | --- | --- | --- | --- | --- | --- | --- | --- | --- | --- | --- | --- |
|  | **Individual 1** | **Individual 2** | **Individual 3** | **Individual 4** | **Individual 5** | **Individual 6** | **Individual 7** | **Individual 8** | **Individual 9** | **Individual 10** | **Individual 11** | **Individual 12** | **Individual 13** | **Individual 14** | **Individual 15** | **Individual 16** | **Individual 17** | **Individual 18** | **Individual 19** | **Individual 20** |
| Araneae | 0 | 0 | 0 | 0 | 0 | 0 | 0 | 0 | 0 | 0 | 0 | 0 | 0 | 0 | 0 | 0 | 0 | 1 | 0 | 0 |
| Blattodea | 0 | 1 | 0 | 0 | 1 | 0 | 0 | 0 | 0 | 0 | 0 | 0 | 0 | 0 | 0 | 0 | 0 | 3 | 0 | 0 |
| Coleoptera | 0 | 0 | 0 | 0 | 0 | 0 | 0 | 0 | 0 | 0 | 0 | 0 | 0 | 0 | 1 | 0 | 0 | 0 | 0 | 0 |
| Diptera | 2 | 1 | 1 | 1 | 2 | 0 | 5 | 1 | 5 | 1 | 1 | 0 | 2 | 2 | 3 | 0 | 2 | 0 | 4 | 1 |
| Hemiptera | 0 | 0 | 0 | 1 | 0 | 0 | 0 | 0 | 0 | 0 | 0 | 0 | 0 | 1 | 0 | 0 | 0 | 1 | 1 | 0 |
| Hymenoptera | 0 | 0 | 0 | 3 | 0 | 0 | 0 | 0 | 1 | 0 | 0 | 0 | 0 | 0 | 0 | 0 | 0 | 0 | 0 | 1 |
| Lepidoptera | 5 | 23 | 38 | 6 | 23 | 17 | 18 | 20 | 21 | 12 | 2 | 24 | 28 | 14 | 26 | 10 | 5 | 10 | 23 | 24 |
| Mantodea | 0 | 0 | 0 | 1 | 0 | 0 | 0 | 0 | 1 | 0 | 0 | 0 | 0 | 0 | 0 | 0 | 0 | 0 | 0 | 0 |
| Neuroptera | 0 | 1 | 0 | 0 | 0 | 0 | 0 | 0 | 0 | 0 | 0 | 0 | 0 | 0 | 0 | 0 | 0 | 0 | 0 | 0 |
| Nestedness | 0.89 | 0.3 | 0.53 | 0.79 | 0.26 | 0.6 | 0.42 | 0.4 | 0.2 | 0.7 | 1.0 | 0.37 | 0.05 | 0.58 | 0.10 | 0.84 | 0.95 | 0.68 | 0.16 | 0.00 |
| Numbers of consumed orders | 2 | 4 | 2 | 5 | 3 | 1 | 2 | 2 | 4 | 2 | 2 | 1 | 2 | 4 | 2 | 1 | 2 | 4 | 3 | 3 |
| Number of consumed MOTUs | 7 | 26 | 39 | 12 | 26 | 17 | 23 | 21 | 28 | 13 | 3 | 24 | 30 | 17 | 30 | 10 | 07 | 15 | 28 | 25 |

| **Table S3.** Consumption of MOTUs from different arthropod orders by individuals at the MOTU threshold 94% of *Pteronotus mesoamericanus* in the dry forests of Costa Rica. Cell values represent the number of Molecular Operational Taxonomic Units (MOTUs) consumed per individual. Nestedness, number of orders and total number of MOTU consumed are indicated. |
| --- |

|  | Individual 1 | Individual 2 | Individual 3 | Individual 4 | Individual 5 | Individual 6 | Individual 7 | Individual 8 | Individual 9 | Individual 10 | Individual 11 | Individual 12 | Individual 13 | Individual 14 | Individual 15 | Individual 16 | Individual 17 | Individual 18 | Individual 19 | Individual 20 |
| --- | --- | --- | --- | --- | --- | --- | --- | --- | --- | --- | --- | --- | --- | --- | --- | --- | --- | --- | --- | --- |
| Araneae | 0 | 0 | 0 | 0 | 0 | 0 | 0 | 0 | 0 | 0 | 0 | 0 | 0 | 0 | 0 | 0 | 0 | 1 | 0 | 0 |
| Blattodea | 0 | 4 | 0 | 0 | 1 | 0 | 0 | 0 | 0 | 0 | 0 | 0 | 0 | 0 | 0 | 0 | 0 | 6 | 0 | 0 |
| Coleoptera | 0 | 0 | 0 | 0 | 0 | 0 | 2 | 0 | 0 | 0 | 0 | 0 | 0 | 1 | 0 | 0 | 0 | 0 | 0 | 0 |
| Diptera | 2 | 1 | 1 | 1 | 3 | 0 | 4 | 1 | 5 | 1 | 1 | 0 | 5 | 2 | 3 | 0 | 2 | 0 | 6 | 2 |
| Hemiptera | 0 | 0 | 0 | 1 | 0 | 0 | 0 | 0 | 0 | 0 | 0 | 0 | 0 | 1 | 0 | 0 | 0 | 1 | 1 | 0 |
| Hymenoptera | 0 | 0 | 0 | 3 | 0 | 0 | 0 | 0 | 1 | 0 | 0 | 0 | 0 | 0 | 0 | 0 | 0 | 0 | 0 | 1 |
| Lepidoptera | 5 | 25 | 16 | 4 | 21 | 21 | 13 | 20 | 17 | 7 | 2 | 31 | 26 | 16 | 22 | 13 | 6 | 15 | 26 | 34 |
| Mantodea | 0 | 0 | 0 | 1 | 0 | 0 | 0 | 0 | 1 | 0 | 0 | 0 | 0 | 0 | 0 | 0 | 0 | 0 | 0 | 0 |
| Neuroptera | 0 | 1 | 0 | 0 | 0 | 0 | 0 | 0 | 0 | 0 | 0 | 0 | 0 | 0 | 0 | 0 | 0 | 0 | 0 | 0 |
| Nestedness | 0.95 | 0.05 | 0.58 | 0.79 | 0.32 | 0.47 | 0.63 | 0.42 | 0.68 | 0.89 | 1.00 | 0.26 | 0.16 | 0.52 | 0.21 | 0.74 | 0.84 | 0.37 | 0.10 | 0.00 |
| Numbers of consumed orders | 2 | 4 | 2 | 5 | 3 | 1 | 3 | 2 | 4 | 2 | 2 | 1 | 2 | 4 | 2 | 1 | 2 | 4 | 3 | 3 |
| Number of consumed MOTUs | 7 | 31 | 17 | 10 | 25 | 21 | 15 | 21 | 24 | 8 | 3 | 31 | 31 | 20 | 25 | 13 | 8 | 23 | 33 | 37 |

| **Table S4.** Consumption of MOTUs from different arthropod orders by individuals at the MOTU threshold 96% of *Pteronotus mesoamericanus* in the dry forests of Costa Rica. Cell values represent the number of Molecular Operational Taxonomic Units (MOTUs) consumed per individual. Nestedness, number of orders and total number of MOTU consumed are indicated. |
| --- |

|  | Individual 1  Individual 1 | Individual 2 | Individual 3 | Individual 4 | Individual 5 | Individual 6 | Individual 7 | Individual 8 | Individual 9 | Individual 10 | Individual 11 | Individual 12 | Individual 13 | Individual 14 | Individual 15 | | Individual 16 | | Individual 17 | | Individual 18 | Individual 19 | Individual 20 | |
| --- | --- | --- | --- | --- | --- | --- | --- | --- | --- | --- | --- | --- | --- | --- | --- | --- | --- | --- | --- | --- | --- | --- | --- | --- |
| Araneae | 0 | 0 | 0 | 0 | 0 | 0 | 0 | 0 | 0 | 0 | 0 | 0 | 0 | 0 | 0 | 0 | | 0 | | 1 | | 0 | 0 |  |
| Blattodea | 0 | 4 | 0 | 0 | 1 | 0 | 0 | 0 | 0 | 0 | 0 | 0 | 0 | 0 | 0 | 0 | | 0 | | 8 | | 0 | 0 |  |
| Coleoptera | 0 | 0 | 0 | 0 | 0 | 0 | 3 | 0 | 0 | 0 | 0 | 0 | 0 | 1 | 0 | 0 | | 0 | | 0 | | 0 | 0 |  |
| Diptera | 2 | 1 | 2 | 1 | 4 | 0 | 9 | 1 | 7 | 1 | 1 | 0 | 7 | 2 | 3 | 0 | | 2 | | 0 | | 4 | 4 |  |
| Hemiptera | 0 | 0 | 0 | 2 | 0 | 0 | 0 | 0 | 0 | 0 | 0 | 0 | 0 | 1 | 0 | 0 | | 0 | | 1 | | 1 | 0 |  |
| Hymenoptera | 0 | 0 | 0 | 3 | 0 | 0 | 0 | 0 | 2 | 0 | 0 | 0 | 0 | 0 | 0 | 0 | | 0 | | 0 | | 0 | 1 |  |
| Lepidoptera | 6 | 31 | 15 | 7 | 29 | 31 | 18 | 21 | 19 | 12 | 2 | 35 | 30 | 25 | 24 | 17 | | 5 | | 17 | | 22 | 54 |  |
| Mantodea | 0 | 0 | 0 | 1 | 0 | 0 | 0 | 0 | 1 | 0 | 0 | 0 | 0 | 0 | 0 | 0 | | 0 | | 0 | | 0 | 0 |  |
| Neuroptera | 0 | 1 | 0 | 0 | 0 | 0 | 0 | 0 | 0 | 0 | 0 | 0 | 0 | 0 | 0 | 0 | | 0 | | 0 | | 0 | 0 |  |
| Nestedness | 0.90 | 0.05 | 0.74 | 0.79 | 0.21 | 0.26 | 0.37 | 0.58 | 0.68 | 0.84 | 1.00 | 0.16 | 0.10 | 0.32 | 0.47 | 0.63 | | 0.95 | | 0.53 | | 0.42 | 0.00 |  |
| Numbers of consumed orders | 2 | 4 | 2 | 5 | 3 | 1 | 3 | 2 | 4 | 2 | 2 | 1 | 2 | 4 | 2 | 1 | | 2 | | 4 | | 3 | 3 |  |
| Number of consumed MOTUs | 8 | 37 | 17 | 14 | 34 | 31 | 30 | 22 | 29 | 13 | 3 | 35 | 37 | 29 | 27 | 17 | | 7 | | 27 | | 27 | 59 |  |


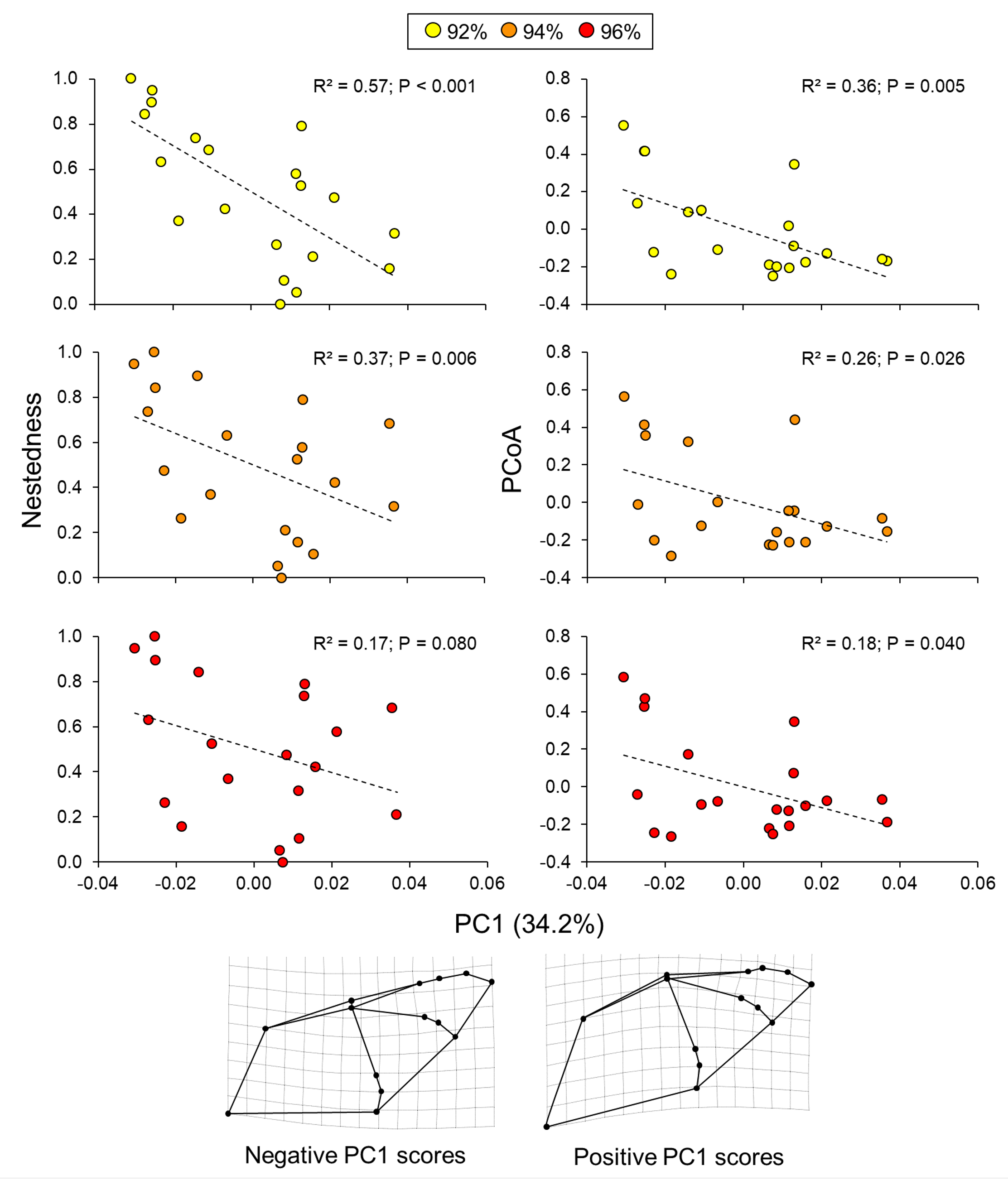


**Figure S1.** Relationship between wing shape and diet (nestedness and similarity [PCoA]) of *Pteronotus mesoamericanus* for different MOTU thresholds (92%, 94%, and 96%). The wing shape representations below the x-axis represent the extrapolated twofold values of the lowest (left inset) and highest (right inset) PC1 scores. Low scores represent wings with a more triangular shape while high scores represent a more rounded wing. The value in parenthesis indicates the proportion of the total wing shape variance; r² and P-values is indicated according to the partial correlation obtained in the multiple regression analysis (global adjusted r² = 0.60 for nestedness and global adjusted r² = 0.40 for PCoA; see results for more details).





**Figure S2.** Relationship between wing shape and diet (nestedness and similarity [PCoA]) of *Pteronotus mesoamericanus* for different MOTU thresholds (92%, 94%, and 96%). The wing shape representations below the x-axis represent the extrapolated twofold values of the lowest (left inset) and highest (right inset) PC2 scores. Low scores represent wings with a more triangular shape while high scores represent a more rounded wing. The value in parenthesis indicates the proportion of the total wing shape variance; r² and P-values is indicated according to the partial correlation obtained in the multiple regression analysis (global adjusted r² = 0.60 for nestedness and global adjusted r² = 0.40 for PCoA; see results for more details).


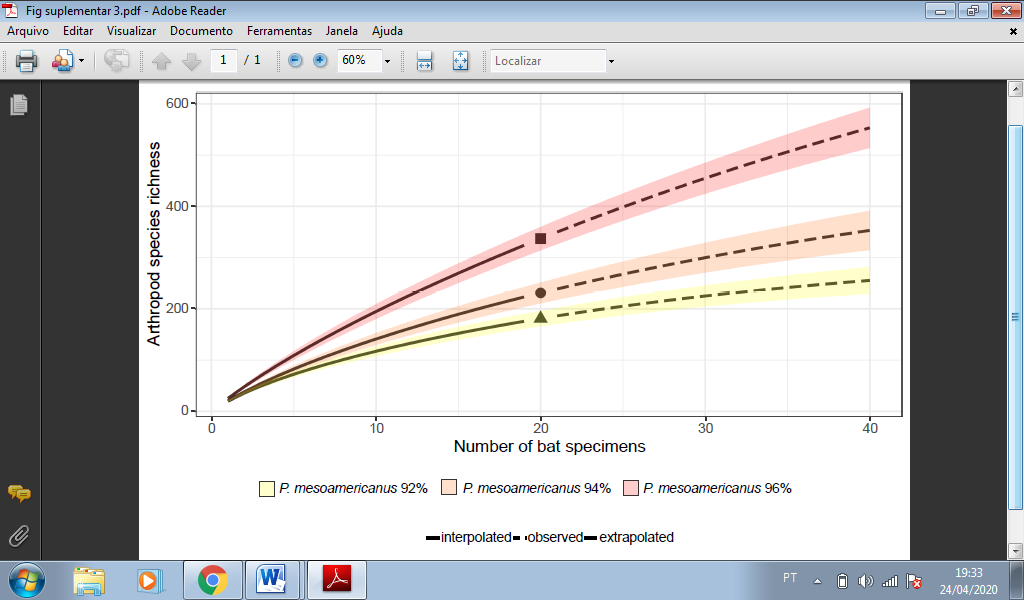


**Figure S3.** Individual-based rarefaction curves estimating the species richness of arthropods present on the diet of the bat species *Pteronotus mesoamericanus* for different MOTU thresholds (92%, 94%, and 96%) during the wet season in the dry forest of Sector Santa Rosa (of ACG) (2015). Red line indicates the richness extrapolating 3 times the number of faecal samples analysed for the present bat species.
